# Photocage-initiated time-resolved solution X-ray scattering investigation of protein dimerization

**DOI:** 10.1107/S2052252518012149

**Published:** 2018-09-13

**Authors:** Inokentijs Josts, Stephan Niebling, Yunyun Gao, Matteo Levantino, Henning Tidow, Diana Monteiro

**Affiliations:** a The Hamburg Center for Ultrafast Imaging, University of Hamburg, Hamburg 22761, Germany; b The Department of Chemistry, The University of Hamburg, Hamburg 20146, Germany; c The Department of Physics, The University of Hamburg, Hamburg 20355, Germany; d The Max Planck Institute for the Structure and Dynamics of Matter, Hamburg 22761, Germany; e ESRF, The European Synchrotron, Grenoble 38000, France; f The Department of Physics and Chemistry, The University of Palermo, Palermo, Italy

**Keywords:** biophysics, X-ray solution scattering, photocaging, structural biology

## Abstract

This work demonstrates a new method for investigating time-resolved structural changes in protein conformation and oligomerization *via* photocage-initiated time-resolved X-ray solution scattering by observing the ATP-driven dimerization of the MsbA nucleotide-binding domain. Photocaged small molecules allow the observation of single-turnover reactions of non-naturally photoactivatable proteins. The kinetics of the reaction can be derived from changes in X-ray scattering associated with ATP-binding and subsequent dimerization. This method can be expanded to any small-molecule-driven protein reaction with conformational changes traceable by X-ray scattering where the small molecule can be photocaged.

## Abstract 2

Photocaging in combination with X-ray solution scattering allows for the time-resolved study of protein dynamics in solution. This method is versatile and allows for accurate triggering of protein function.

## 1. Introduction   

During the course of their function, proteins undergo a series of structural transitions, sampling different structural intermediates. Therefore, the relationship between protein structure and its function is determined by its dynamic properties (Levantino *et al.*, 2015 ▸). The timescales of appearance and decay of these intermediates span multiple orders of magnitude – from femtoseconds to seconds – and all contribute to the overall efficient and specific function of the protein. Equilibrium measurements provide a picture of an ensemble of all the thermodynamically accessible protein conformational states and are dominated by the lowest energy (highest populated) structures. Determining the structure of protein functional intermediates is not trivial as the system has to be perturbed from its non-equilibrium state and followed in real time. Time-resolved experiments allow for such information to be gathered. Here, we focus on time-resolved X-ray solution scattering (TR-XSS), a technique that provides real-time global structural information on the protein during its functioning. Specifically, we target non-reversible non-natural photoactivatable protein reactions by coupling TR-XSS with photocaging strategies.

TR-XSS has been used to follow numerous biological processes since it can provide direct structural information on the species present as reactions progress (Levantino *et al.*, 2015 ▸). However, TR-XSS requires a trigger in the form of either photoexcitation, a temperature jump, mixing of ligands or a pH jump to initiate the reaction as uniformly as possible in the biomolecule of interest. To our knowledge, pump–probe small-angle X-ray scattering (SAXS) or wide-angle X-ray scattering (WAXS) has not been applied to an irreversible protein reaction pathway, and TR-XSS studies have pre­dominantly focused on photoactive proteins or proteins amenable to pH- or temperature-induced oligomerization or folding/unfolding (Cammarata *et al.*, 2008 ▸; Rimmerman *et al.*, 2017 ▸; Kim *et al.*, 2015 ▸). Light triggering offers some of the fastest and homogeneous reaction-initiation conditions. Considering that only a small fraction of proteins are naturally photoactivatable, non-natural triggering methods have to be employed. Since many protein processes are mediated by small molecules, synthetic analogs of the molecules bearing photocleavable protecting groups can be prepared and used to initiate protein function by photocleaving with a light pulse.

Photocage-initiated TR-XSS is a new experimental approach that allows tracking of time-resolved structural transitions initiated by small-molecule binding using X-ray solution scattering (XSS). The ligand is rapidly released into the protein solution by photocleavage and XSS profiles are collected at appropriate time delays after decaging. Photodecaging of small-molecule ligands offers a path to the observation of single turnover events of proteins that are not naturally photoactivatable, opening up many possibilities for studying reaction kinetics and structural dynamics in a much wider range of biological systems.

**Figure 1:**
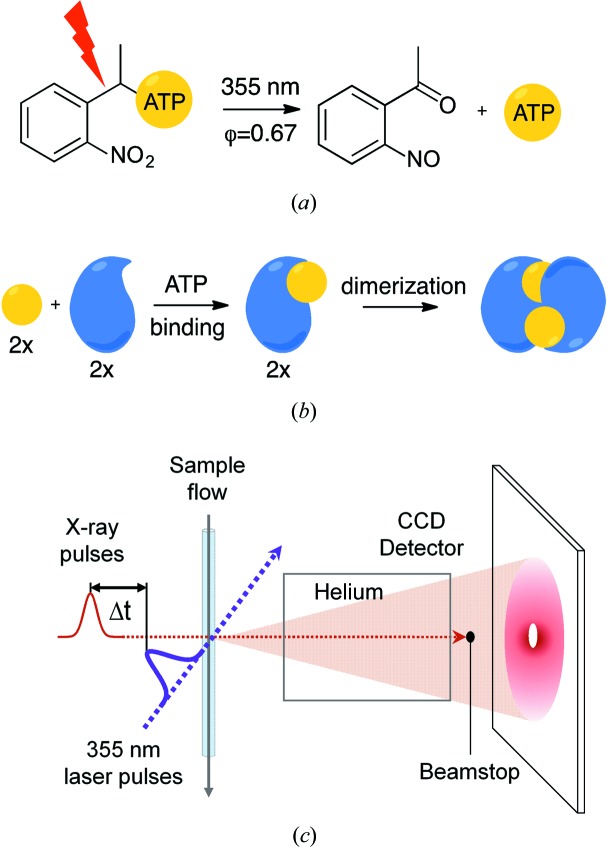
Overview of the experimental setup and protein structural transitions. (*a*) Photoexcited decaging of the NPE group from NPE-ATP using laser irradiation at 355 nm releases free ATP into solution. The decaging reaction happens on a 10 ms timescale. The quantum yield of the reaction is 0.67 (Syberg *et al.*, 2012 ▸). (*b*) Mechanism of ATP-dependent (yellow circle) dimerization of NBDs (blue). (*c*) Schematic diagram of the experimental set up at the beamline.

Adenosine triphosphate (ATP) hydrolysis drives numerous enzymatic reactions and biological processes from the translocation of substances across cell membranes, signaling cascades, protein folding and chaperoning to protein degradation. These enzymes, termed ATPases, utilize the binding and breakdown of ATP as a source of energy to undergo changes in their tertiary or quaternary structure during their functional cycle. Caged ATP has previously been used to study the mechanism of force generation by kinesins as well as the dynamics of ATP-sensitive potassium channels in muscle by patch-clamping and tensile measurements, respectively (Clapp & Gurney, 1992 ▸; Goldman *et al.*, 1982 ▸). In this study, we combine laser-flash photolysis of 1-(2-nitrophenyl)-caged ATP (NPE-ATP) with time-resolved X-ray-scattering measurements to investigate the ATP-dependent dimerization of soluble nucleotide-binding domains (NBDs) from a bacterial lipid flippase, MsbA. These domains are key drivers for the ‘power stroke’ mechanism of substrate translocation in the ATP-binding cassette-transporter family of proteins. The binding of ATP to each of the NBDs induces conformational changes in each domain with the subsequent formation of a closed dimer, while further allosteric changes in the transmembrane domain of the transporter result in the translocation of solutes across the membrane through the alternating access mechanism (Ward *et al.*, 2007 ▸). Time-resolved Fourier transform infrared spectroscopy (TR-FTIR) was previously employed to indirectly investigate the dimerization kinetics of the soluble NBDs from MsbA by ATP-decaging (Syberg *et al.*, 2012 ▸). In contrast, X-ray solution scattering directly reports on changes in protein structure. Here, we explore the strategy of ATP-decaging in combination with X-ray scattering to gain time-resolved structural insight into the dimerization of NBDs. Using the experimental setup outlined in Fig. 1 ▸ we were able to observe the formation of a dimerization-competent conformation of NBDs (present in the very early time-point of the experiment) and the subsequent NBD dimer formation. The ATP-promoted NBD dimer in itself is a metastable intermediate which decays upon hydrolysis of the ATP molecule (∼10 s) and cannot be imaged using the standard static SAXS data-collection strategies. Since our current understanding of the NBD dimer formation comes predominantly from crystallographic models trapped in the post-ATP hydrolysis stages, our method provides an alternative way to structurally visualize short-lived protein states in solution.

## 2. Materials and methods   

### 2.1. Protein expression and purification   

The sequence for the MsbA nucleotide-binding domain (NBD, residues 337–582) from *Escherichia coli* was amplified by PCR and cloned into the pneK vector under the control of the T7 promoter with an N-terminal His_6_-tag and a TEV protease cleavage site using standard protocols. Protein overexpression was carried out in *Escherichia coli* BL21(Gold) cells in Terrific broth medium at 37°C, expression was induced with 0.2 m*M* IPTG (OD_600_ = 1) followed by incubation at 20°C for 16 h. Overexpressed NBD protein was purified to homogeneity using immobilized nickel-affinity chromatography. Purified protein was dialyzed against 20 m*M* Tris, 200 m*M* NaCl, 5 m*M* MgCl_2_, frozen in liquid nitro­gen and stored at −80°C.

### 2.2. Experimental setup   

The experiment was carried out at beamline ID09 at the European Synchrotron Radiation Facility (ESRF, Grenoble, France). ID09 provides polychromatic X-rays centered at 15 keV with a 3% bandpass in a 100 × 60 µm (*H* × *W*) full width at half-maximum (FWHM) focused beam at the sample position. A Rayonix MX170-HS detector was used at a distance of 300 mm to the sample. A helium cone with a 1.6 mm diameter beamstop was placed between the sample and the detector. The sample-to-beamstop distance was 210 mm. For this experiment, 15 µs X-ray pulses were used.

A 0.5 m*M* protein solution with 1.5 m*M* NPE-caged ATP {adenosine, 5′-(tetrahydrogen triphosphate), P′′-[1-(2-nitrophenyl)ethyl] ester disodium salt, Jena Bioscience} was flowed continuously through a 1 mm diameter quartz capillary using a Miniplus 3 peristaltic pump (Gilson). The sample was photolyzed using a 355 nm ns laser (Vibrant from Opotek, Carlsbad, CA, USA, sold by Quantel, Les Ulis, France) with 4 mJ per 5 ns pulse and a 1.7 × 0.2 mm (*H* × *W*) FWHM. The relative timing of the laser and X-ray pulse was controlled electronic­ally (laser–X-ray jitter <5 ps). At this wavelength, NPE-ATP has an extinction coefficient of 430 *M* ^−1^ cm^−1^. The low extinction coefficient allows for uniform penetration of the laser through the sample. The laser power was calculated to deliver ∼4 photons per caged ATP. NPE-ATP has a quantum yield of 0.6 at a photolysis wavelength of 360 nm, yielding a final concentration of ∼0.95 m*M* ATP post-photolysis (McCray *et al.*, 1980 ▸).

At *t*_0_, the laser pulse for photoexcitation of the sample was delivered and after the desired time delay, the photoexcited sample volume was probed by the X-ray pulse. The maximum possible time delay was calculated from the flow velocity and the area of overlap between the laser and the X-ray pulses, to guarantee that only photoexcited sample was measured. After each X-ray probe pulse, the sample flowed continuously to ensure full refreshment of the laser-interaction volume. Depending on the flow-rate, this step determined the maximum repetition rate of the measurement. The laser-pump, X-ray-probe and sample refresh cycle was repeated multiple times and multiple X-ray pulses (20–100) were integrated onto the detector before readout to increase the signal-to-noise ratio. After the detector readout, the time delay was changed and the cycle repeated. Multiple images were collected for each time delay and merged during data processing to further increase the signal-to-noise ratio. Non-photoexcited data were collected with a negative time delay between the pump-laser and the X-ray probe pulse (−100 µs). Photoexcited and non-photoexcited images were interleaved to keep the experimental conditions between dark and light data sets as similar as possible. Further details on the experimental setup are summarized in Fig. S1 and, in conjunction with Table S1, specify the exact experimental parameters (including flow rates and maximum repetition rates) for each data set collected.

### 2.3. Data reduction   

The normalization factor for each exposure was calculated using the sector integration of the total counts within the radial range of 74.0–83.4 pixels. Appropriate normalized buffer profiles were subtracted. The background-corrected images were then azimuthally averaged. The resulting profiles for each time delay were averaged to acquire a sufficient signal-to-noise ratio.

For each measurement series (up to four time points including dark shots), the single shots were grouped by delay time and further processed. To avoid deviations attributed to occasional air bubbles, single shots were rejected if the integrated signal in the range between 0.05 and 2.5 Å^−1^ was outside the range of the median ±1 s.d. (standard deviation). One exception was the measurement series with the time-points (50, 100 and 200 ms), for which a tighter criterion of median ±0.05 s.d. was used because of the presence of an air bubble in certain frames. In total, 7% of all shots were rejected. To obtain difference scattering curves, shots with the same delay times were averaged and the average of the dark shots from the same measurement series were subtracted.

### 2.4. Calculation of NBD-ATP and NBD-AMPPCP SAXS curves and *ab initio* modeling   

Theoretical scattering curves were calculated with *CRYSOL* (Svergun *et al.*, 1995 ▸) using the available high-resolution X-ray structures of the NBDs in the apo, ADP, AMPPCP-bound and dimeric states [PDB codes, 5dgx (Halavaty *et al.*, unpublished work); 5idv (Mayclin *et al.*, unpublished work); 3b60 (Ward *et al.*, 2007 ▸)]. The radius of gyration (*R*_g_) at each time delay was obtained using the indirect Fourier transform implemented in *GNOM* (Svergun, 1992 ▸) using the scattering curve over the *q* range 0.04–0.3 Å^−1^. For the calculation of the dimer difference curves, the scattering was computed up to a *q* value of 1 Å^−1^ and scaled in the high *q* range. The *ab initio* modeling was conducted using *DAMMIN* (Svergun, 1999 ▸). A total of 30 independent *DAMMIF* (Franke & Svergun, 2009 ▸) runs were performed to generate the initial structure pool. The final filtered dummy-atom model was then calculated by averaging the initial structures with *DAMAVER* (Volkov & Svergun, 2003 ▸).

### 2.5. Singular value decomposition and second-order kinetic fits   

For the singular value decomposition (SVD), the difference scattering curves in the *q* range between 0.038 and 0.5 Å^−1^ were used. Two significant components were identified and, as judged from the transpose of the *V* matrix, the first component was assigned to the dimeric species.

Assuming a reaction equation of the type 2*A*→*B*, where *A* is the NBD monomer and *B* is the NBD dimer, the rate of dimerization (*k*_dim_) was calculated by fitting the change in radius of gyration between 50 ms and 1.4 s using the integrated second-order rate equation, 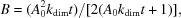
where *A*_0_ is the initial concentration of monomeric NBD (0.5 m*M*).

### 2.6. Cumulative first-ranked singular-values correlation map   

A comparison between the absolute SAXS curves was performed in the same *q* range as that used for the difference curves. The metric of similarity *m*_*i*,*j*_ is defined as the weight of the first-ranked singular value, 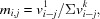
where *v* ^*k*^_*i*−*j*_ is the *k*-ranked singular value of the mean centering matrix of the scattering profiles (*I*_*i*_, …, *I*_*j*_) according to the consecutive time delay. The color assignment of the *m*_*i*,*j*_ is done by utilizing the ‘Reds’ colormap in the *matplotlib* package. The white color maps the lowest *m* value (*m* = 0.433) and the darkest red maps identity (*m* = 1).

### 2.7. Software   

*Pyfai* implemented scripts were used for data reduction and radial integration. Self-written Python programs using the packages *numpy* and *scipy* (Jones *et al.*, 2001 ▸) were used for processing the scattering spectra, including singular value decomposition. Kinetic fitting was performed using *Prism7* (GraphPad).

## 3. Results and discussion   

In order to follow the dimerization of NBDs using X-ray scattering by decaging NPE-ATP, we used a 5 ns 355 nm laser pulse which, in our experimental setup, was overlapped with the X-ray pulses in the sample capillary (Figs. 1 ▸ *c* and S1). The achievable time-resolution of the experiment is limited by the decaging time of NPE-ATP (∼10 ms) following flash photolysis (McCray *et al.*, 1980 ▸). A slow-flowing liquid-delivery system was employed to provide automatic refreshment of the sample between each pump–probe pulse while keeping the laser-illuminated volume in the X-ray path (see Fig. S1 of the Supporting Information).

Time-resolved scattering data were collected in a pump–probe setup with a time delay between the laser-induced photolysis and incident X-rays from 50 ms to 1.4 s, and over a *q* range of 0.038–2.5 Å^−1^. The shortest time delay (50 ms) was selected to be longer than the known NPE-ATP decaging time (McCray *et al.*, 1980 ▸) as the aim of the experiment was to observe structural changes related to the slow dimerization step, not the nucleotide binding event (Syberg *et al.*, 2012 ▸). It is worth noting that the full enzymatic mechanism involves not only the binding of ATP followed by dimerization, but also a much slower ATP hydrolysis step (∼10 s) which results in the dissociation of the dimerized NBDs. The duration of this slow step was longer than the available time-delay window at beamline ID09 (ESRF) at the time of this experiment.

Scattering from the unexcited (dark) state of the protein was recorded at a negative temporal offset (−100 µs) between the laser and X-ray pulses. The resulting radius of gyration (*R*_g_) of 21.5 ± 0.19 Å of the dark state calculated from the absolute scattering fits well with the theoretical *R*_g_ of 20.7 Å, derived from the crystal structures of MsbA NBD in the apo state [PDB codes, 5idv; 3b5w (Ward *et al.*, 2007) ▸]. In contrast, the crystallographic dimer (PDB code, 3b60) has a larger theoretical *R*_g_ of 23.5 Å. The experimental *R*_g_ indicates that the presence of caged-ATP does not promote dimerization of the MsbA NBD.

**Figure 2:**
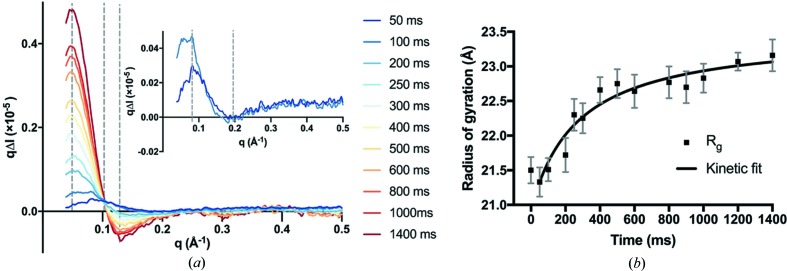
Time-resolved SAXS data following photo-decaging of NPE-ATP. (*a*) Plot of the scattering difference curves (*q*Δ*I* *versus* *q*) calculated by subtracting the protein scattered with a negative laser offset (−100 µs) from all the subsequent time-point measurements. Inset shows the difference curves of the first two time points (50 and 100 ms). (*b*) Radius of gyration representing NBD dimerization and kinetic fit with an increase in *R*_g_ over time (black squares with corresponding standard deviations). The time points after laser excitation were fitted with a second-order kinetic function (black line). The calculated rate constant for dimerization (*k*_dim_) from the fit is 6200 *M* ^−1^ s^−1^.

Although absolute scattering is used to measure the overall scattering volume, difference curves are valuable in tracing local and specific conformational changes in the protein. Hence, to observe the structural changes in the NBDs that result from ATP binding and dimerization after decaging, difference curves were plotted by subtracting the dark (apo) state curves from the subsequent time delays. The scattering difference curves shown in Fig. 2 ▸ reveal an evolution of the difference signal predominantly at a low *q* range (0.038–0.5 Å^−1^). A clear and consistent increase in the forward scattering intensity (when *q* < 0.1 Å^−1^) is easily noticeable, which is assigned to the formation of the ATP-bound NBD dimer (Fig. 2 ▸ *a*). We find that the change in the determined *R*_g_ is consistent with an increase in protein size (Fig. 2 ▸ *b*). The final *R*_g_ of 23.16 ± 0.23 Å at the 1.4 s time point matches well with that of the crystallographic dimer.

As the increase in *R*_g_ represents the structural change associated with the dimerization of the NDB, a second-order kinetic rate equation for the model of 2*A*→*B* (*A* is the NBD monomer and *B* is the dimer) can be fitted. The fit is shown in Fig. 2 ▸(*b*) and yields a rate constant of 6200 *M* ^−1^ s^−1^ for the dimerization step (*k*_dim_). The kinetics presented by Syberg *et al.* (2012 ▸) were approximated with multiple exponential global fits, rather than the more biochemically representative bi­molecular kinetic model presented here, making it impossible to directly compare the kinetic constants. For comparison purposes only, a single exponential fit to our *R*_g_ data yields a rate constant of 2.4 s^−1^ which is similar to the rate constant of 1.4 s^−1^ obtained by Syberg *et al.* (fit not shown).

Calculated difference curves between the crystallographic dimer and monomer (PDB codes 3b60 and 5idv) show features similar to those visible in our 1400 ms time-delay difference curve at *q* ranges <0.13 Å^−1^ (Fig. S2). Additionally, the computed *ab initio* molecular envelopes of the monomeric and dimeric NBDs also closely resemble their respective crystal structures (Fig. S3). Altogether, these similarities advocate that decaging of NPE-ATP induces NBD dimerization.

Previously reported dimerization kinetics of NBDs obtained by FTIR spectroscopy relied on spectral changes of the nucleotide signal (Syberg *et al.*, 2012 ▸). Time-resolved spectroscopy is a well established technique that yields information on the local environment of the probe and provides an indirect correlation with the structural changes in the protein. In contrast, time-resolved X-ray scattering yields direct structural information of the protein during its conformational cycle. Our TR-XSS experiments allow direct visualization of the ATP-induced structural transitions in the NBDs as well as their oligomerization. Specifically, at the first time point (50 ms), positive and negative peaks at *q* values of 0.08 and 0.2 Å^−1^, respectively, are already present, indicating fast structural transitions within the NBD molecules during the dead time of the experiment. These early structural changes can be correlated with the binding of ATP to the NBD, and were not captured in the previous TR-FTIR work, highlighting the importance of global structural information. Further structural changes are visible, as the negative peak at *q* = 0.2 Å^−1^ broadens between 100 and 300 ms, and from 400 ms onwards, a sharper peak emerges at *q* = 0.13 Å^−1^. These structural changes appear to be a single transition event with an isosbestic point at 0.1 Å^−1^.

**Figure 3:**
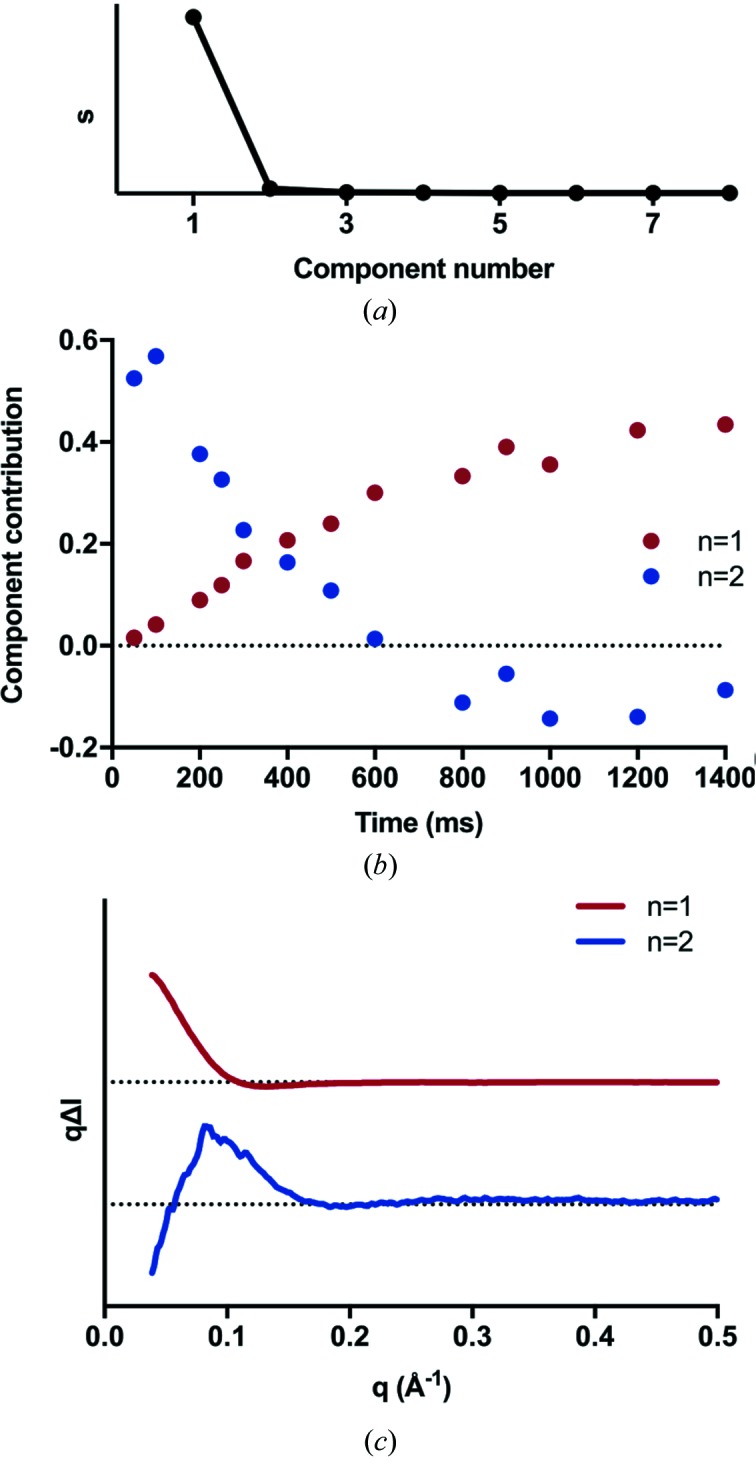
Single-value decomposition analysis of the TR-XSS data reveals the presence of two components. (*a*) Singular values (from the diagonal of matrix s) plotted on a linear scale. (*b*) Singular (V^T^) matrix showing the relative amplitudes of the eigenvectors (component 1 and 2 in red and blue, respectively) *versus* time. (*c*) Singular (U) matrix showing the eigenvectors for the two major components.

SVD analysis of the difference scattering curves was used to interrogate the structural transitions further. The analysis indicates the presence of one major and one minor component (Fig. 3 ▸ *a*), where the first component is by far the most significant. We attribute this component to the NBD dimer owing to its continuous increase in concentration over the measured time points (Fig. 3 ▸ *b*), consistent with the increase in *R*_g_. The high significance of this component is not surprising as it is the dominating structural change expected for the long time delays probed during this study.

SVD analysis also shows a second minor component, which is already present at 50 ms and decays rapidly. We attribute this component to a nucleotide-capture event by the NBD and conformational changes necessary to yield a dimerization-capable state. We speculate that the scattering difference curve at 50 ms represents one of the intermediates relating to the nucleotide-bound conformation of the NBD domain, as it correlates well with the calculated scattering curves from the crystal structures of NBDs in complex with various nucleotides (PDB codes, 5dgx and 3b60; Fig. S2). Specifically, both the experimental difference curves and the calculated difference curves from the nucleotide-bound crystal structures present a positive peak at 0.08 Å^−1^ and a negative contribution at 0.2 Å^−1^. Even within the two nucleotide-bound crystal structures, the calculated XSS curves show slight differences in this region, indicating that changes in this *q* range are internal structural rearrangements in response to the nucleotide binding. In-depth characterization of this binding step would require scattering earlier time points with faster ATP-decaging analogs. This would allow us to model the kinetics of this step and better determine the populations present in the sample; however, this is outside the scope of this investigation.

To further strengthen our study and corroborate our findings from the SVD analysis, we performed an independent analysis by using a cumulative first-ranked correlation map of the absolute scattering curves (Fig. S4). This correlation map shows continuous structural transitions, revealing fast-decaying states in the first 100 ms, followed by a steady evolution of intermediate states, which are particularly visible between 200 and 800 ms time delays.

Our ability to track the dimerization of the NBDs in real-time by combining the photoexcited decaging of ATP and TR-XSS offers new possibilities for the investigation of structural dynamics for numerous ATP-dependent reactions, and potentially in any photo-decageable system. The ability to study non-equilibrium and unidirectional protein function coupled to structure opens up new possibilities to obtain invaluable insight into molecular structure–function relationships. Since other NPE-caged bioactive ligands such as GTP are also commercially available, this approach opens the door to study the mechanistically relevant conformational transitions in reactions such as G-protein-coupled receptor signaling. Caged ligands with varying photocaging groups can be synthesized, allowing for tunability of decaging wavelengths, quantum yields and decaging rates, at the same time offering time resolutions varying from nanoseconds to milliseconds. Coupled with TR-XSS, this technique paves the way for the investigation of tertiary and quaternary structural changes in proteins triggered by ligand binding. Several parameters must be taken into consideration for a successful TR-XSS experiment in order to overcome the technical difficulties associated with conducting these experiments. Most importantly, the signal-to-noise ratio and the magnitude of the difference signal in the *q* range expected for the structural change under investigation has to be detectable in order for a clear structural and kinetic model to be extracted. For example, for large proteins and protein complexes undergoing local structural perturbations, the difference in signal would be small compared with the overall scattering power of the protein. Therefore, high-resolution and high signal-to-noise images must be acquired, requiring either brighter X-ray sources or longer total accumulated exposure times. This caveat is being overcome by the emergence of very bright synchrotron sources as well as X-ray free-electron lasers and developments in detector technology. These advancements allow for these techniques to be used more generally.

## Supplementary Material

Supporting information, table and figures.. DOI: 10.1107/S2052252518012149/mf5027sup1.pdf
