# Supplementary material for: Photocage-initiated time-resolved solution X-ray scattering investigation of protein dimerization
Source: IUCrJ. 2018 Sep 13;5(Pt 6):667–72. doi: 10.1107/S2052252518012149 (PMC6211537; doi:10.1107/S2052252518012149)
Supplement: Supplementary file 1 [file m-05-00667-sup1.pdf]

IUCrJ

Volume 5 (2018)

Supporting information for article:

Photocage-initiated time-resolved solution X-ray scattering investigation of protein dimerization

Inokentijs Josts, Stephan Niebling, Yunyun Gao, Matteo Levantino, Henning Tidow and Diana Monteiro

Table S1 Parameters for data collection

Dataset number	Time delay (Δt, ms)	Flow velocity (mm/s)	X-ray pulses/image	number of images for each Δt	Data acquisition rate
1	-0.1, 600, 800, 1000	0.02	20	23	0.1 Hz
2	-0.1, 50, 100, 200	0.20	100	25	1.0 Hz
3	-0.1, 300, 400, 500	0.10	80	20	0.5 Hz
4	-0.1, 250	0.10	40	20	0.5 Hz
5	-0.1, 900, 1200, 1400	0.02	20	8-9	0.1 Hz

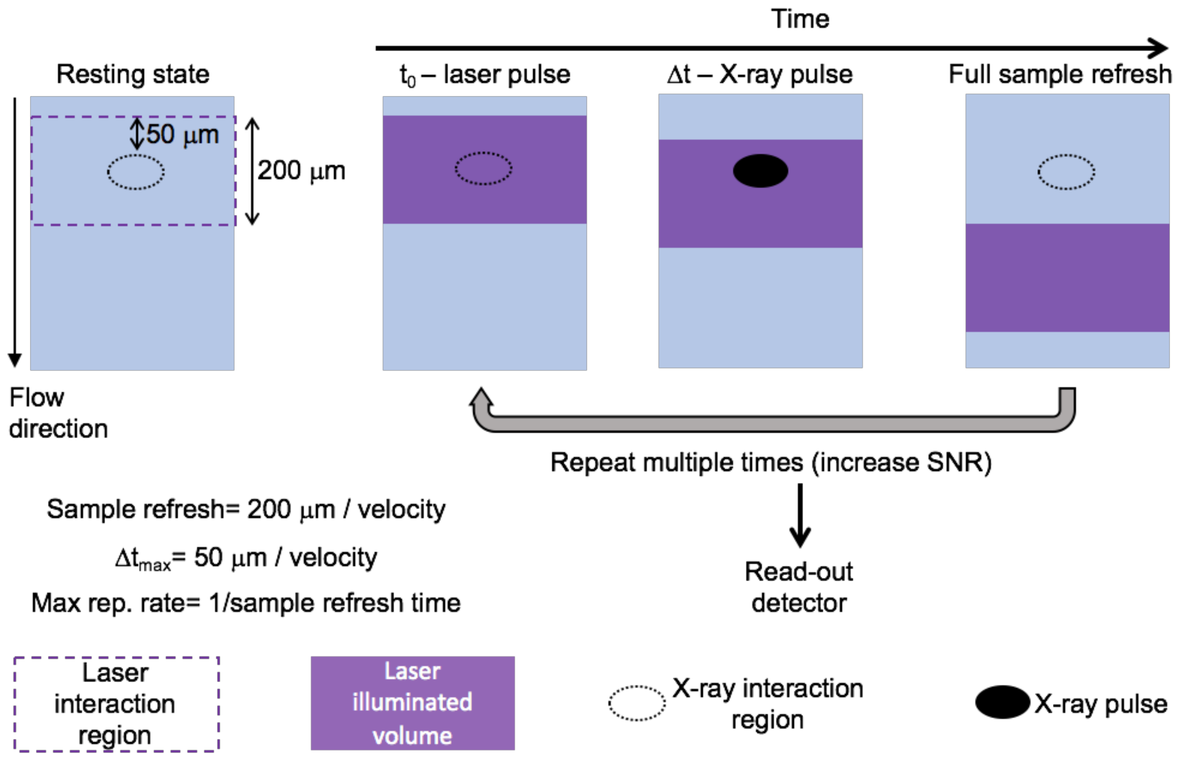

Figure S1 Flow scheme for illumination, probing and refreshing of the sample. The X-ray and laser interaction regions are centered and aligned to the capillary. The resting state shows the overlap of the pump and the probe. The X-ray spot is 0.10x0.06 mm and the laser 1.7x0.2 mm (HxV, FWHM). At t_0, the laser pulse illuminates the sample. After a specified time-delay, the X-ray pulse hits the activated sample volume. The sample is allowed to flow for a full 200 μm before the measurement is repeated to ensure full sample refresh. The pump-probe-refresh cycle is repeated multiple times, accumulating several X-ray pulses on the detector to increase the signal-to-noise ratio before the detector is read out. After the image is read out, a new time delay is

set and the cycle repeated. The sample was allowed to flow a maximum of 50 μm before the X-ray probe pulse arrives to ensure the X-rays probe a laser-illuminated sample volume.

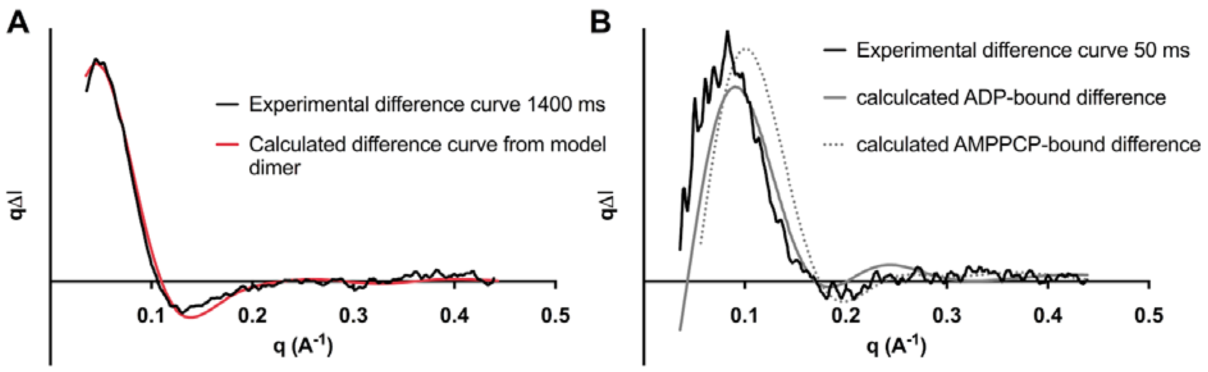

Figure S2 A) Difference curves between the calculated dimer and the 1400 ms time-delay are in agreement with each other. B) A good agreement between calculated difference XSS curves from crystal structures of the NBD monomer bound to ADP (PDB 5DGX), non-hydrolysable ATP analogue (AMPPCP, PDB 3B60) and 50 ms time-delay difference curve. All the curves show positive differences centered at 0.08 Å^{-1} as well as a decrease in intensity at 0.2 Å^{-1}. Mild deviations between the two calculated curves at these scattering angles indicate these structural changes are correlated to the nucleotide binding region.

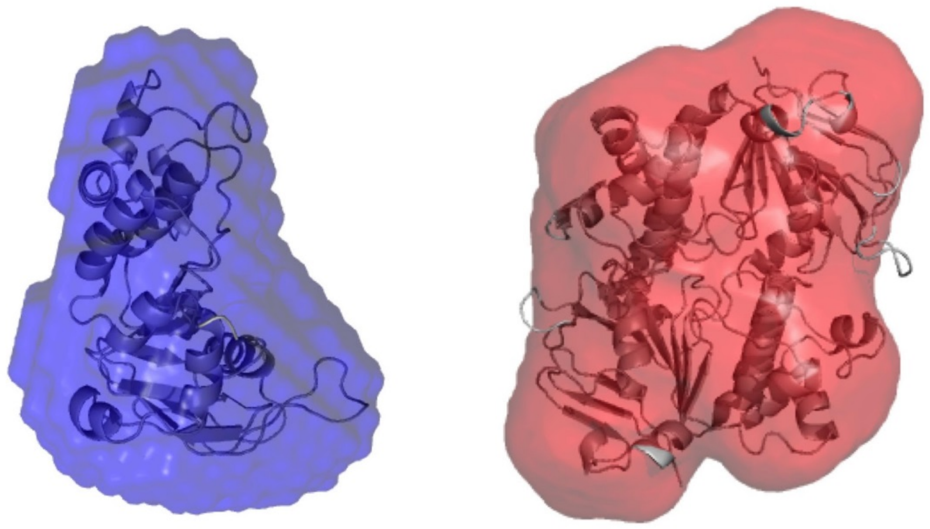

Figure S3 *Ab initio* models of the dark state (blue) and of 1400 ms data point (red) superimposed with monomeric (PDB: 5IDV) and dimeric NBD (PDB: 3B60) from MsbA respectively.

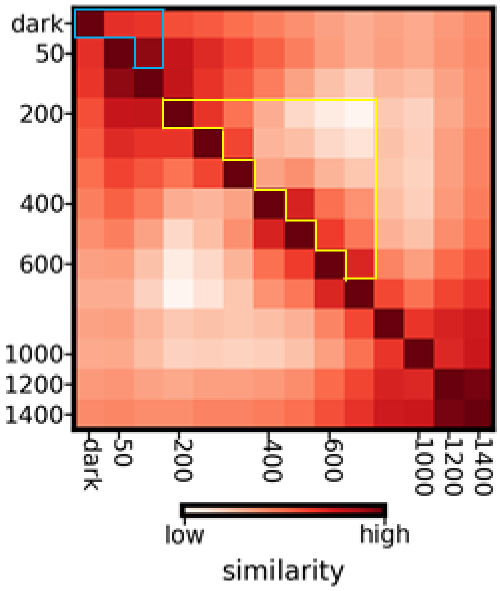

Figure S4 Cumulative first-ranked singular values correlation map based on the absolute XSS data. Sudden drop of scattering profile similarity suggests fast structural transitions after the decaging (blue zone). Significant difference was observed from 200 ms to 800 ms as an indication of binding-related dimerization (yellow zone).
